# Science, Names Giving and Names Calling: Change NDM-1 to PCM

**DOI:** 10.4103/0973-1229.77446

**Published:** 2011

**Authors:** Ajai R. Singh

**Affiliations:** Editor, MSM.

**Keywords:** *Names giving*, *Eponymous names*, *NDM-1*, *PCM*, *Superbug*, *Carbapenems*, *Plasmids*, *Metallo-β-lactamase*, *Journal editors*, *Submission guidelines*, *Names changing*, *Scientific names giving*

## Abstract

A journal editor recently apologised for publishing a 2010 paper in which authors designated an enzyme as New Delhi metallo-β-lactamase-1 (NDM-1) and its related gene bla_NDM-1_ after a city, New Delhi. This name had raised an outcry in India, with health authorities, media and medical practitioners demanding New Delhi be dropped from the name. The name was actually first given in another 2009 paper, whose corresponding author remains the same as the 2010 paper. There is a tradition of eponymous names in science. But those found derogatory to races, groups, cities, and countries have been changed. For example, “Mongolism” was changed to Down’s syndrome; “Australia” antigen to HBsAg; “Mexican” Swine flu to H1N1; “GRID” (Gay Related Immune Deficiency) and 4H-Disease (Haitians, Homosexuals, Haemophiliacs and Heroin Users Disease) to AIDS. It is necessary that NDM-1 also be changed to a name based on scientific characteristics. NDM-1 must be changed to PCM (plasmid-encoding carbapenem-resistant metallo-β-lactamase). It is also necessary to review the tradition of naming organisms, diseases, genes, etc. after cities, countries and races. Often, such names giving amounts to names calling. It needs to be discarded by scientists in all new names giving from now on. “Geographical” and “racial” names giving must be replaced by “scientific” names giving. Journal editors must ensure that such scientific names giving is laid down as standard guideline in paper submissions. All such names still in currency need to be phased out by replacing them with names based on scientific characteristics, or in honour of their pioneering scientist/s or institutions. The lead author of the above 2010 paper has said he was not consulted about the final draft and did not agree with the conclusions of the paper. To ensure that corresponding authors do not ride roughshod over co-authors, and lead and other authors do not backtrack on papers, editors must ensure written concurrence of all authors, especially the lead author, to the final draft of a paper and include this in their guidelines for paper submissions.

## Introduction

A 12 Jan 2011 news report says the editor of a fellow journal has tried to assuage hurt feelings by apologising and accepting that it was an error of judgement to name a “superbug” after a city (see http://timesofindia.indiatimes.com/india/Lancet-says-sorry-for-Delhi-bug-/articleshow/7261135.cms)(Sinha, 2011).

The issue had earlier appeared to die down after some heated debate from both sides. This apology from the editor seems to have racked up issues, with both sides raising the same objections and making the same claims.

Scientists, health authorities and politicians from India were understandably incensed when the paper in question (by Kumaraswamy *et al*., 2010), was first published online in August 2010, since the enzyme [called New Delhi metallo-β-lactamase-1 (NDM-1)] was “designated” after the capital of the country, New Delhi. The Indian Health Ministry and some prominent Indian medical practitioners, as also parliamentarians, had then alleged that it was a means to malign the country and its booming medical tourism industry (Pandey, 2010; Donnelly, 2010; Indo-Asian News Service, 2010; IANS, 2010; Sharma, 2010; Narayan, 2010). They also alleged that this paper, and such other methods, were means by which some countries were trying to rein in their own dwindling patient population seeking cost-effective treatment in India. From the authors’ side, some maintained it was sound science, and also common practice to name organism after cities (Walsh *et al*., 2010).

The editor in the URL above also maintains that although the naming was unfortunate, the science was sound. Moreover, it was up to the scientists concerned to change the name.

The present paper takes it further, stressing that even if the scientists concerned take no action, science should, when it is convinced such action is needed.

### Four important issues

Opinion may be divided whether the editor did well to so apologise. One can also debate the editor’s motives and intentions for saying what he did at this time, which can be a happy hunting ground for some. But that is not the purpose here.

We, at *MSM*, are concerned since this incident raises four important issues:

- The science and evidence regarding the so-called name “NDM-1”
- Fundamental questions about names giving in science
- Whether a change of name is appropriate in the present case
- What journal editors need to ensure in future

## I. The Science and Evidence Regarding the So-called “NDM-1”

The 12 Jan 2011 news item referred to above said the editor of the *Lancet* has apologised for an error of judgement by naming a “superbug” after New Delhi.

Let us first have the facts. The editor had not so named it. That was the name given in a paper. Also, the paper in question was published in *The Lancet Infectious Diseases*, not in *The Lancet*. These are two sister publications of Elsevier, a Reed Elsevier Company, whose editors also happen to be different. Then, how come *The Lancet* editor owned up responsibility for something published in a sister journal? Although not the editor, The *Lancet* editor is also the “Publisher/Editorial Director” of *The Lancet Infectious Diseases*, and has thus rightfully spoken on its behalf.

This paper was published in Sept 2010 [online in August 2010], and has authors with scientific credentials from all over the globe [see Table 1.]

The lead or first author is from Chennai, India, as are five others, who are from Haryana, Kerala, Kolkata, Chennai and Varanasi (Marked** in Table 1). There are two authors from Pakistan too, one each from Karachi and Lahore, and also, one each from Sweden and Australia. There are also authors from the UK.

It was a multicentric collaborative effort with authors holding scientific credentials drawn from various institutions in India, Pakistan, Sweden, Australia and the UK published in a proper speciality journal, with the lead author and four others from India.

The science seems to be based on solid grounds. Moreover, it is the Indian lead author, and co-authors, who should be held responsible for the New Delhi name, if at all.

Also note that the designated name, NDM-1, after New Delhi was *used* here, true, but not *given* by this paper. It was already in use in microbiological studies. So let us not find fault where none lies.

Do we, then, give this paper a clean chit? Scientific study by qualified people, whose lead author himself was an Indian, and with four other authors from India, published in a peer-reviewed journal of some standing, which did not originally name it after New Delhi at all, but only continued using a name already given by someone else?

What are some people in India making a big brouhaha about maligning India and targeting its medical tourism industry?

Just wait a moment.

### Some more facts

We have still not analysed all the evidence available.

Look at the authors’ list. This study has, guess what, 31 co-authors [See Table 1]! A record of sorts. There are 19 authors from the UK, 2 from the School of Medicine, Cardiff University; and (mark this) *17 authors from the Health Authorities in UK* – 16 from the Health Protection Agency Centre for Infections, London, UK, and 1 from the Northumbria Healthcare NHS Foundation Trust, presumably also a health authority related agency (all marked ^c^ and ^k^ in Table 1).

**Table 1:** The Sept 2010 *Lancet Infect Dis*. paper details

Journal
The *Lancet Infectious Diseases*, 2010, September, 10(9), p597–602. doi: 10.1016/S1473-3099(10)70143-2.
Paper
Emergence of a new antibiotic resistance mechanism in India, Pakistan, and the UK: A molecular, biological, and epidemiological study
Authors
Karthikeyan K Kumarasamy,^a^ Mark A Toleman,^b^@ Timothy R. Walsh,^b^*@ Jay Bagaria,^c^ Fafhana Butt,^d^ Ravikumar Balakrishnan,^c^ Uma Chaudhary,^e^ Michel Doumith,^c^ Christian G Giske,^f^@ Seema Irfan,^g^ Padma Krishnan,^a^ Anil V Kumar,^h^ Sunil Maharjan,^c^ Shazad Mushtaq,^c^ Tabassum Noorie,^c^ David L Paterson,^i^ Andrew Pearson,^c^ Claire Perry,^c^ Rachel Pike,^c^ Bhargavi Rao,^c^ Ujjwayini Ray,^j^ Jayanta B Sarma,^k^ Madhu Sharma,^e^ Elizabeth Sheridan,^c^ Mandayam A Thirunarayan,^l^ Jane Turton,^c^ Supriya Upadhyay,^m^ Marina Warner,^c^ William Welfare,^c^ David M Livermore,^c^ and Neil Woodford^c^
Author Affiliations
^a^Department of Microbiology, Dr ALM PG IBMS, University of Madras, Chennai**, India
^b^Department of Infection, Immunity and Biochemistry, School of Medicine, Cardiff University, Cardiff, UK
^c^Health Protection Agency Centre for Infections, London, UK
^d^Department of Microbiology, Shaukat Khanum Cancer Hospital, Lahore, Pakistan
^e^Department of Microbiology, Pandit B D Sharma PG Institute of Medical Sciences, Haryana**, India
^f^Department of Clinical Microbiology, Karolinska University Hospital, Stockholm, Sweden
^g^Department of Pathology and Microbiology, The Aga Khan University, Karachi, Pakistan
^h^Department of Microbiology, Amrita Institute of Medical Sciences, Kerala**, India
^i^University of Queensland Centre for Clinical Research, University of Brisbane, Herston, QLD, Australia
^j^Department of Microbiology, Apollo Gleneagles Hospital, Kolkata**, India
^k^Department of Medical Microbiology, Northumbria Healthcare NHS Foundation Trust, Tyne and Wear, UK
^l^Department of Microbiology, Apollo Hospitals, Chennai**, India
^m^Department of Microbiology, Institute of Medical Sciences, Banaras Hindu University, Varanasi**, India

* **Corresponding author (common to both papers in Tables 1 and 2)**

@ Common authors in both papers in Tables 1 and 2.

** Authors from Indian centers

Such a large number of authors from the UK Health Authorities, 17 for a single paper, by their sheer numbers, must have ensured their viewpoint prevailed, in spite of there being 5 authors from India. One can understand how they managed to protect their health industry interests, and that of their citizens, incidentally maligning the Indian medical tourism industry in the process.

The corresponding author (marked * in Table 1) is from the Department of Infection, Immunity and Biochemistry, School of Medicine at Cardiff University, who probably got all the authors to collaborate with him. He is named third author here, but must have played a key role, as will be obvious in the next section. For, we have to look into some more facts available to piece the whole story together.

### The study responsible

Let us trace the roots of this designated name, NDM-1. And here, we come to an interesting phenomenon.

The name was first used in a Dec 2009 paper (online Sept 2010) by Yong and colleagues (Yong *et al*., 2009) published in *Antimicrobial Agents and Chemotherapy*, another proper speciality journal (of the American Society for Microbiology) [see Table 2].

**Table 2:** The Dec 2009 *Antimicrob Agents Chemother* paper details

Journal
*Antimicrob Agents and Chemotherapy*, 2009 December, 53(12), p5046—5054. Published online 2009 September 21. doi: 10.1128/AAC.00774-09.
Paper
Characterization of a new metallo-β-lactamase gene, bla_NDM-1_, and a novel erythromycin esterase gene carried on a unique genetic structure in *Klebsiella pneumoniae* sequence type 14 from India
Authors
Dongeun Yong,^1,2^ Mark A. Toleman,^2^@ Christian G. Giske,^3^@ Hyun S. Cho,^4^ Kristina Sundman,^5^ Kyungwon Lee,^1^ and Timothy R. Walsh^2^*@
Author Affiliations
Yonsei University College of Medicine, Research Institute of Antimicrobial Resistance, Seoul, Republic of Korea,^1^;
Department of Medical Microbiology, Cardiff University, Cardiff, United Kingdom,^2^;
Clinical Microbiology, MTC—Karolinska Institutet, Karolinska University Hospital, Stockholm, Sweden,^3^;
Yonsei University College of Life Science and Biotechnology, Seoul, Republic of Korea,^4^;
Department of Clinical Microbiology, Örebro University Hospital, Örebro, Sweden^5^

* Corresponding author (common to both papers in Tables 1 and 2)

@ Common authors in both papers in Tables 1 and 2.

The genesis of the name is as follows. Yong *et al*. 2009 reported of a 59-year-old patient of Indian origin settled in Sweden. He had uncontrolled type 2 diabetes mellitus and had earlier had multiple strokes. While on a visit to India in 2007, he developed gluteal abscess and was treated with limited success in Haryana as also in a hospital at New Delhi. He shifted to Sweden where he continued treatment. Here, in January 2008, his urine sample tested positive for *Klebsiella pneumoniae* harbouring a new enzyme of the metallo-β-lactamase family.

Since this patient had earlier reportedly been in a New Delhi hospital, the sample was “designated” New Delhi metallo-β-lactamase, or NDM-1, probably by the microbiologist/s there, “1” probably being the number of the sample.

### The interesting part

Look a little carefully at Table 2. You will find that three out of the seven colleagues in this 2009 paper happen to be none other than authors from the 2010 *The Lancet Infectious Diseases*(marked, ’ @ Common authors in both papers in Tables 1 and 2’).

*And the corresponding author for both papers is the same* (marked “*corresponding author common to both papers in Tables 1 and 2”), along with another author from Cardiff, and the one from Karolinska, Sweden (who was probably responsible for the Swedish connection and the patient sample).

Also, the method of using author names for publication in this 2009 paper is the same as in the 2010 *The Lancet Infectious Diseases* paper. The lead author here is from the Republic of Korea, as are some others, just as the lead author in *The Lancet Infectious Diseases* paper was from India, as were some others.

In other words, the lead authors in both the 2009 and 2010 papers were the fronts while the control probably remained with the corresponding author in both cases.

This, incidentally, is what the lead author of *The Lancet Infectious diseases* study has also implied when he disclosed he was not consulted as regards the final draft, and did not agree with the alarm raised in the conclusions there – that those visiting India beware, for medical tourism or otherwise (Narayan, 2010; see http://timesofindia.indiatimes.com/city/chennai/Indian-author-says-superbug-report-is-fudged/articleshow/6302479.cms),because their health would be severely compromised in a country with poor asepsis and “high level of antibiotic resistance” (Yong *et al*., 2009). For, this is what the lead author is quoted as saying in the above press report: “I do not agree with the last paragraph which advises people to avoid elective surgeries in India. While I did the scientific work, correspondence [sic] author… of Cardiff University was assigned to edit the report”. Contrary to this is the statement from the section “Contributors” in the Kumaraswamy *et al*., 2010 paper: “All authors were involved in the compiling of the report and approved the final version”.

This means either the lead author is concealing the truth or the final draft was edited and prepared by the corresponding author and sent away to the editorial office, and accepted as approved by all authors at that office, without verification, in good faith, since the lead and other authors must have earlier given the corresponding author a blanket permission to do everything regarding the paper on their behalf.

Can anyone imagine a paper getting published without the concurrence of the lead author? Someone from the editorial department at *The Lancet Infectious Diseases* must answer for this. Either the corresponding author had ridden rough shod, or the lead author was backtracking to reduce the domestic heat on him. More on this later, in the last section on editorial responsibilities.

In other words, the main actor in this whole naming act remained the corresponding author, who continued to hold the threads, while the rest played to his tune.

### Designated, and therefore, liable to change

To be fair to them all, Yong *et al*.’s 2009 paper, which named the original sample, called it “designated” NDM-1. Even *The Lancet Infectious Diseases* 2010 paper calls it “designated” NDM-1.

This means it was only so “designated” in both these papers. Designated means subject to confirmation/acceptance by others, which means there is a definite scope for change.

### What happens to subsequent publications?

The legitimate question that comes up here is: If one even thinks of name change, what happens to papers published subsequently, since the name must have already been used in numerous papers published later?

Conducting a literature survey, one finds that papers which have subsequently appeared have been quick to latch on to this name [Muir and Weinberg, 2010; Centres for Disease Control and Prevention (CDC), 2010; Karthikeyan *et al*., 2010; Deshpande *et al*., 2010; Sidjabat *et al*., 2011; Bonomo, 2011; Poirel *et al*., 2010; Poirel *et al*., 2011a; Poirel *et al*., 2011b; Poirel *et al*., 2011c; Nordmann *et al*., 2011a; Nordmann *et al*., 2011b; Pillai *et al*., 2011; Samuelsen *et al*., 2010a; Samuelsen *et al*., 2010b;Woodford *et al*., 2010; Rodriguez-Martinez *et al*., 2010]. This is understandable as scientists usually do so unmindful of the repercussions of names giving, obsessed as they are with the technicalities of their work. What is understandable, however, is not necessarily justifiable.

Now, on closer scrutiny, one finds that most of the papers using this name have the same authors as the Yong *et al*., 2009, and Kumaraswamy *et al*., 2010 papers; and the rest are a small close circle of authors. Moreover, this name has been in use only from late 2009 to early 2011, i.e. less than 18 months.

So, it is not a fact that *numerous* biochemical/microbiological scholars have used the name rampantly for *many* years.

The rest of them who have used the name are the interested lay press and internet encyclopedias (e.g. Wikipedia, 2011), who would be quick to latch on to a new name as well, provided the broad scientific community agrees.

So, it is not the case that a scientific name change, if necessary, will cause a big reorganisation of the scientific record.

### What is scientifically ominous about this enzyme and related gene?

As we are at the science involved, we must also understand why the interest in scientific circles in this enzyme and related gene. To understand this, we must know what is ominous about this enzyme. I will try and simplify it as much as possible. It belongs to the family of carbapenemases, which cause antibiotic resistance to carbapenems, one of the highest classes of antibiotics. Carbapenems are a special class of antibiotics used when the standard line fails. If they fail, one is left with very little option, i.e. only the polymixins (like colistin) and tigecycline. In fact, as late as the 1980s and 1990s, carbapenems were considered the “last resort antibiotics” used primarily against extended-spectrum β-lactamase (ESBL) or AmpC-producing gram-negative bacteria (Bonomo, 2011).

Here was a development whereby one more microbial mechanism was defeating man’s war against infectious diseases. What was further discomforting was that this enzyme caused genetic changes in bacteria whereby they could transfer immunity to other bacteria via plasmids. Plasmids are DNA type substances that can be passed on via horizontal transfer to other organisms, thus conferring immunity against higher forms of antibiotics. And not just to *K*. pneumoniae, but many others, rather the whole of the Enterobacteriaceae family (some as benign as *Escherichia coli* normally present in our intestines). Also, such horizontal plasmid transfer was potentially possible to other bacteria types as well. In other words, this genetic transfer could potentially make even the most benign organism the most virulent. This has not still happened, but it held such a potential threat.

Bacteria and their hosts (human beings) are in a never-ending battle. But this was one development that would give bacteria a clear edge, something science could ill afford to ignore.

The whole uproar and interest in biochemical/microbiological circles to understand this, and control it, is therefore, most legitimate.

*Plasmid encoding* as the important characteristic of this gene and *carbapenem resistance* as an important characteristic of this enzyme must be noted, for it has importance in our later discussion about a scientific name change.

### Spread and positive fallout

Also noteworthy is another fact. Although the earlier paper found it in the UK, India, Pakistan, Bangladesh, etc., later reports have located it also in Ontario, Canada (CTV.ca News Staff, 2010; White, 2010); Japan (AFP/wk, 2010); USA [Centers for Disease Control and Prevention (CDC), 2010; McNeil Jr., 2010; Smith, 2010)]; Sultanate of Oman (Poirel, 2011b); Kenya (Poirel, 2011c); Norway (Samuelsen, 2010b); and Belgium (AFP, (2010). Reports from a Mumbai centre have also found it in a hospital there (Deshpande *et al*., 2010), and UK put forth an alert as early as 2009 itself (Health Protection Agency, 2009). So, its universal presence is disconcerting, to say the least.

Equally noteworthy is a positive fallout of this in India, since some have suggested a re-look at the rampant misuse of antibiotics there (Abdul Ghafur, 2010), and in other such countries, and called for a revamp of the medical prescription system, as also the need to be less cheesed with names giving after the city, New Delhi (Chatterjee, 2010). Also worthy of note is work in developing newer antibiotics to combat such infections (Alazraki, 2010; Carroll, 2010).

While its universal presence is cause for concern, work on developing newer antibiotics and revamping systems in India and such other countries can be heartening fallouts.

Let us, however, continue with the main point about names giving in science, which is the focus of this communication.

## II. Fundamental Questions about Names Giving in Science

This section will focus on fundamental questions about what is involved in scientific names giving, whether name changes have ever occurred, and in what circumstances. For this, we must try and answer two questions related to names giving in science:

1. Is it standard practice in science to name organisms/diseases after cities/scientists?
2. Is it a practice to be deplored?

### Scientific names giving

#### 1. On appearance, characteristics or properties

It is not the case that there is anything wrong with a scientist naming a disease, gene, virus or bacteria based on their appearance or characteristics. Bacteria, for example, have been named according to:

- Their appearance, e.g. *Staphylococcus* (from Greek: σταφυλή, *staphylē*, “bunch of grapes” and κόκκος, *kókkos*, “granule”) and *Streptococcus* (from Greek: στρεπτος *streptos*, meaning “easily bent” or “twisted”, like a “chain” (twisted chain)];
- Their disease connections, e.g. pneumococcus (coccus causing pneumonia); and
- Their staining properties, e.g. gram positive or gram negative.

This appears perfectly legitimate, and there seems to be no offence meant (unless of course bacteria, or their lobby of supporters, object to them being called a bunch of grapes!).

#### 2. On scientists

The convention of names giving after scientists also exists. Scientists/philosophers/philosophies have been associated with names of

- Bacteria (e.g. Koch’s bacillus, *Escherichia coli*);
- Diseases (e.g. Hansen’s Disease, Korsakoff’s Psychosis, Alzheimer’s disease, Klinefelter’s Syndrome, Huntington’s chorea, Parkinson’s disease);
- Psychological tests [e.g. Rorschach’s Ink-blot test, Wechsler’s Adult Intelligence Scale (WAIS)];
- Laboratory solutions/stains [e.g. Fehling’s solution, Benedict’s solution, Tollen’s reagent, Gram’s method, etc.];
- Brain areas (e.g. Broca’s area, Wernicke’s area, Broadmann’s areas, etc.);
- Neurotransmitter principles (e.g. Dale’s principle);
- Therapies (e.g. Beck’s Cognitive therapy, Heimlich’s manoeuvre, Adler’s Individual psychology, Epley’s manoeuvre, etc.);
- Processes, theories and movements (e.g. Brownian movement, Newton’s Laws of motion, Maslow’s hierarchy of needs, Einstein’s theory of general relativity, Freudian psychoanalysis, Pavlov’s classical conditioning, etc.);
- Oaths (e.g. Hippocratic oath); and
- Therapies based on philosophies (e.g. Zen psychotherapy, Existential psychotherapy).

But all these were done because we believe scientists would feel proud to be so named. In fact, it is an honour in science to have a disease or bacterium named after a scientist. I wonder if any scientist, or their heirs, would have allowed it if they felt it derogatory. I wonder if we would have done it if it were considered derogatory in the first place.

### Why the problem?

While a name can be appropriately connected to a scientist, place or institution, it can also be thoughtlessly so connected. The problem arises due to naming a gene, disease, bacterium, enzyme, etc. after a city/country, which some scientist presumes to be the city/country of its origin. It is the latter that we got to be wary of, and this has got highlighted in the present case. Names like “German” measles, “Australia” antigen, “Mexican” swine flu, “Delhi” belly, “Mongolism”.

Think about it this way. What is *German* about German measles? Or *Australian* about Australia antigen? Or *Mexican* about H1N1? Or *Delhi* about the so-called “Delhi belly”? Or “Mongol” about Mongolism? Or Haitian about the 4-H disease (the earlier name of AIDS, for those who are not aware)? All these are universally present conditions, but so named just because some scientist presumed they originated there, or some condition mimicked some race.

Graphic, true, but indiscreetly used words, since a convention allows for it (for there is one of using toponymous words for diseases). Why indiscreet? Because it can amount to names calling, unless the city/country is proud to own responsibility. If Germany, Australia, Mexico, New Delhi, Haiti and the Mongols felt proud of being named in such a way, could anyone object? But they do not. That is the key issue, and this incident has only italicised it.

It is time scientists stopped being naïve to names giving in science.

### Names changing

The question that now comes up is whether a name, once given, can be changed. The answer is yes. Whenever names have been considered derogatory to a certain place or community, science has been persistent that the names be changed.

We just saw Down’s syndrome was earlier called “Mongolism”, Rubella was “German” measles, and HBsAg (Hepatis B surface antigen) was “Australia” antigen. Traveller’s diarrhoea was earlier called “Delhi” belly, and schizophrenia was earlier called “dementia” praecox. A patient with a psychosis was earlier called “insane”, “mad”, “lunatic”, etc. The “pandemic H1N1/09” (WHO) or “2009 H1N1 flu” (US Center for Disease Control) was earlier called “Mexican” swine flu. AIDS was earlier GRID (Gay Related Immune Deficiency) in the lay press, and even a scientific body like the US CDC earlier called it the “4-H disease” (the “Haitians”, “homosexuals”, “haemophiliacs”, and “heroin” users disease). And scientists did not consider it bad form when such names were first used, otherwise they would never have used it in the first place. Now they do, don’t they?

What was the essential reason why these name changes took place? It was to prevent science from, knowingly or unknowingly, indulging in names calling.

So, there are two conventions. One is naming after cities and countries. The second one is changing such names if it hurts a nation’s or race’s sensibilities. It is this other convention that concerns us here. Also, all name changes that have occurred have been based on their scientific characteristics, or in honour of a scientist. To the question “What’s in a name” (Nataraj, 2010), the answer is lots, if it has to be scientific. And to the contention, we avoid ersatz patriotism in science (Chatterjee, 2010), we need science in names giving to pre-empt precisely such ersatz patriotism.

There is a tradition to eponyms. True. But they were mainly used because they were a convenient tool to categorise an entity at a time when science and medicine lacked the tools to understand the underlying reasons and mechanisms behind diseases, bacteria, syndromes, etc. As an interim arrangement, and till we had the scientific wherewithal, it was a convenient designation. Now when we do have such means, eponyms must be avoided and consigned to the dustbin of medical history, unless it is an honour to institute it in some person, institution or country’s name. For example, if someone found the definitive treatment for AIDS or malignancy, or the aetiology of Schizophrenia, we would happily name them after that person, institution or even city/country, would we not?

### Why change the name NDM-1?

Barring a few, no one in India is proud to be associated with this name. It smacks of racialism, as it is used to label a city, country and its neighbourhood as harbouring some virulent bacterial strains, as though virulent bacterial strains are not present elsewhere. The Indian Health Ministry has said in no uncertain terms that it does not agree to this enzyme and related gene being named after New Delhi (Donnelly, 2010; IANS, 2010; Indo-Asian News Service, 2010; Sharma, 2010).To the best of my knowledge, barring a single author (Chatterjee, 2010), no other Indian author, scientist or otherwise, has expressed happiness or pride at New Delhi being so named. And the pride is rather tongue in cheek even in that article. Nor would the majority of Indian medical practitioners feel proud of such a name, not to forget those who would be beneficiaries of a name change – the hospitals and consultants in India, who would benefit from the so-called “medical tourism” there.

Make no bones about it. Although I am an Indian and proud of being one, I do not espouse this name change to promote the “medical tourism” industry in India. That will stand or fall on its own merits. Or, bring in my patriotism through the back door. There are better avenues available to manifest it, and although the universality of science does not negate patriotism, it cannot be its happy hunting ground. What concerns me, and should also concern those who care for scientific name giving, is that we should not allow any person, scientist or otherwise, to use the name of any city or state in any nation without considering the sensibilities of its people.

To remedy this, it is best that names giving be based on science, and science alone, *so that our naming is as scientific as the rest of what we produce in our researches*.

A systematic start has to be made to put scientific names giving into practice. So, why not start with India, and the name NDM-1? “Scientific names giving” is one contribution to science, India could justifiably be proud of. No one could possibly object if it were named, e.g. “India’s contribution to scientific names giving”.

Then, to answer the questions raised at the beginning:

1. Is it standard practice in science to name organisms/diseases after cities/scientists?
  There is a convention of this nature, which may be forwarded only if it does not hurt a nation/race’s sensibilities and is meant to honour a scientist/institution.
2. Is it a practice to be deplored?
  Not necessarily, if the point made in the first answer is ensured.

## III. Change NDM-1 to PCM

The so-called “Delhi belly” was changed correctly to Traveller’s diarrhoea. It is time NDM-1, also named after this same Delhi, be changed to something more appropriate and scientific.

What could that be? We could have considered naming it after a scientist or an institution, but there is neither one scientist nor one institution involved that can be given sole credit. So, that is out at least here.

The other is a name based on science. To give us a scientifically appropriate name change, we must understand what is special, and ominous, about this enzyme and gene.

We discussed that earlier. The two factors unique to this metallo-β-lactamase enzyme and related gene are:

- its transfer by plasmids, i.e. *plasmid encoding* and
- its resistance to the higher class of antibiotics, carbapenems, i.e. *carbapenem resistance*.

Hence, the correct name should be *plasmid-encoding carbapenem-resistant metallo-β-lactamase*, in short, PCM.

In other words, PCM should replace NDM-1, both in the enzyme and the gene. The gene in question becomes bla _PCM_ from bla_NDM-1_.

What this takes care of is the following: stopping to hurt a nation’s sensibilities, and doing so by giving a name that is scientific, based on its distinctive characteristics as per our existing state of knowledge. This knowledge may alter over time, but the name will stand scientific testimony to how it all started.

We thus introduce science in names giving and carry out one more change from names calling to names giving. Forwarding, thereby, one more worthy tradition in science: of *changing from “geographical” or “racial” to “scientific” names giving.*

## IV. What Journal Editors Need to Ensure in Future

To understand what journal editors need to ensure, let us try and answer three questions related to this incident and the discussion up till now:

- Should editors learn from this incident and see to it that researchers desist from naming bacteria, diseases, etc. after cities, countries or races, especially because (unintentionally perhaps) scientists may not be sensitive to sensibilities of countries and peoples?
- Will it be a good policy that henceforth, before accepting for publication, editors insist authors change any such names after cities and countries?
- What should editors do, so that corresponding authors ensure concurrence from all authors, especially the lead author?

### The role of an editor: Scientific names giving

Some editors may hold the view that an editor has no role to play in the matter at all; he looks into the science of the paper and there his job ends. It is the scientist who needs to get educated about scientific names giving, if at all, and if he does not do it, popular or peer pressure could be exerted for change. But even to do so or not is not in the domain of an editor’s role. In any case, it is the sole prerogative of the scientist who first so named it to name howsoever he pleases, preferably following a tradition, and change it if he deems fit. It is like we do with our own names. In fact, there may be editors who would defend a scientist’s right to name an organism, gene, disease, howsoever he decides, and such a defence may be considered necessary to forward science. Otherwise, so the argument can go, anyone could start raising objections to names giving under some pretext or the other.

Now, to answer this contention. The job of an editor does not end with the science in a paper. It extents to ensuring that there is science even in names giving in a paper. In fact, *this is part of the science of a paper*.

In other words, an editor must ensure scientific names giving. If an editor ensures this, the need for popular or peer pressure to change names, or for scientists who first so named it to change names later, would just not arise. Prevention is better than cure. While it is necessary to defend a scientist’s right to name organisms, it is equally necessary to ensure that such names are based on science, not on an author’s whims and fancies. After all he is not naming his child or an enemy, to base it on a whim or a prejudice, respectively. Also, the name is not his personal property or domain, to dispose of, name, or alter as he pleases. The moment it is made public through a scientific paper, it enters the public intellectual domain and is the property of science, to be accepted, rejected or altered by fellow scientists. Also, since he is naming an object in science, that naming better be scientific, otherwise it will face censure, and needless arguments between peers, of which one manifestation is this very paper.

Think of it this way. If the editor of the Yong *et al*. 2009 paper had put his foot down and asked that the name after a city be changed to something scientific, the uproar that has followed and the need for papers like this would simply not arise. And scientists would be allowed to do what they do best, science, free from the burden of national and other such regional sensibilities hampering scientific activities. I would myself have been spared the task of unearthing so much evidence to just prove a point. It has been no joy, I can assure you, to find fault with peers. Biochemistry and microbiology are not my forte, either. It was done only to prevent further perpetuation of such mistakes, having realised what was happening.

This is what editors can ensure – preventing further perpetuation of such mistakes by simply rejecting “geographical” and “racial” and insisting on “scientific” names giving, since editors and journals are the primary conduits through which such names reach the public intellectual domain. They cannot absolve themselves of their responsibility under any pretext. When editors are meant to ensure objectivity in the rest of the matter that goes into a paper, how can they not ensure objectivity even in names giving?

Having been blind to this role till now, it is doubly important editors awaken and accept that it would be sound policy for editors to become sensitive to political and racial sensibilities in names giving in science, especially when they may even *appear* to be maligning a city, country, ethnic group, etc.

Moreover, editors cannot afford to immunise themselves from such considerations in the name of furthering science. It really does not further science; it only results in genuine scientific work getting embroiled in needless controversy, as this work did.

Scientists need not carry, or at least need not project, their prejudices about people on to our science. Editors need to be especially careful that this does not occur in the writings published in their journals; for writings may have a limited span, but journals have a legacy, and a reputation to protect, and project.

The Editorial Director of the publication in question has learnt his lesson well. I hope the broader editor community learns it too, before it is forced on to them by repetition of such incidents. His apology has been clear-cut and dignified. I propose editors find better ways to manifest such excellent qualities in the future.

### Name, but not after a city/country

Therefore, Rule Number 1, both for a scientist, and an editor, is:

While a scientist has every right to research anything under the sun, he does not automatically have the right to name it howsoever he chooses, especially if he may appear to be hurting a nation/race’s sensibilities.

A scientist must know the difference between the two. For one right does not automatically ensure the other. But if he does not, an editor must at least know, and take care that the writings published in his journal are not immune to such considerations, as the Editorial Director of *The Lancet Infectious Diseases* realised, albeit belatedly.

To ensure this, Rule Number 2 to be followed by both scientists and editors is:

Names giving should be as scientific as the rest of the published work. By scientific we mean based on scientific characteristics, not on subjectivity, geography or race.

This simple rule just pre-empts all needless controversy and frees science for that it does best – science.

### What do editors and journals need to do to ensure scientific names giving?

What are the modi operandi editors need to adopt?

1. *Have the critical antenna up when you find “geographical” names giving*. Journals and editors must be careful that diseases, bacteria, viruses, etc. named after cities/countries be given a close look, and insist that authors change these if they appear to be derogatory to those cities or countries. First politely, then firmly, if need be – even to the point of rejecting the work, if the researcher becomes adamant.
  It is immaterial what has been accepted practice up till now in the name of toponymous diseases. When editors and journals realise their implications, they must remedy matters.
2. *Allow for names honouring scientists and institutions*. Scientists may, if they wish, name diseases/pathogens after the original scientist/s or the names of their institutions (of course with the institution’s/scientist’s permission, wherever possible).
3. *Encouraging the science does not necessarily extend to defending names giving*. Encourage every *scientific work*, and stick your neck out to support scientists about their science regardless of any opposition. But when it comes to *naming* diseases and organisms, be very sensitive to national sensibilities, and insist that scientists be equally sensitive. This distinction must be made, and legitimately so. Name giving must not amount to, or even *appear* to amount to, names calling.

So Rule Number 3, this time exclusively for editors, is:

Stick your neck out for the science, but wield the stick when you smell names calling.

There is science involved in the research, which must be fiercely protected, but there is no science necessarily involved in name giving, where discretion is the watchword. The fine distinction between name giving and names calling is sometimes unobtrusively crossed by scientists/researchers. Conscientious editors should be especially vigilant that this does not occur in their journals.

## Editor’s role for the corresponding author to obtain concurrence from all authors, especially lead author

As noted earlier, the lead author of *The Lancet Infectious Diseases* study has disclosed that he was not consulted as regards the final draft and did not agree with the alarm raised in the conclusions there that those visiting India should beware (Narayan, 2010). Here was probably the case of a paper getting published without the concurrence of the lead author and, as said earlier, someone from the editorial department at *The Lancet Infectious Diseases* must be answerable for this.

Either the corresponding author had ridden rough shod or the lead author was backtracking to reduce the domestic heat on him. While editors cannot be concerned with the latter, they must be very concerned that they pre-empt the former.

What can editors do to ensure the integrity of the scientific record and also ensure that they do not get embroiled in such controversies?

The following is already being done. Most editorial departments take in writing from all co-authors who the corresponding author would be, and take it he/she has been conferred all rights by the other authors. This is good and useful as a standard policy. While this is necessary for editors to protect their skin, it is not sufficient to protect the scientific record.

The present incident teaches us that in the final check-list before a paper is published:

- It is necessary as a rule for editors/editorial departments to confirm from the corresponding author that all other authors have read and agreed to the *final draft* of the paper before its publication, so that no one can either backtrack or ride rough shod.
- As a double check, it would also be a sound policy to send the *final draft* of the paper to all co-authors and confirm from them *in writing* that they agree to it, and neither the editor nor the co-authors simply trust the corresponding author in the matter. To reduce editorial load and publication delay, the corresponding author may be entrusted the role of expediting such consent, but the paper must be sent to all co-authors by the editorial department.
- If this is not possible for any exceptional reasons (coma, death, criminal sentencing, etc. of a co-author), concurrence from at least the lead author (if he/she is not the corresponding author) and other authors that the corresponding author has complied with this is mandatory before the final draft is accepted for publication. The responsibility is then equally shared between the two important role players in a paper – the lead author and the corresponding author – with the other authors having a full knowledge of what is happening.

While it does involve a little more work by the editorial department, it ensures that journals immunise themselves from potential nefarious activities of corresponding authors who are not lead authors and it is an important step in maintaining the integrity of the scientific record.

### Answering the questions raised earlier

To answer, then, the questions raised at the beginning of this section:

1. Should editors learn from this incident, and see to it that researchers desist from naming bacteria, diseases, etc. after cities, countries or races, especially because (unintentionally perhaps) scientists may not be sensitive to sensibilities of countries and peoples?
  Yes. Editors must take up this role and avoid shirking their responsibility in the name of forwarding science and researcher’s rights to name an entity howsoever they please. They must ensure “scientific” names giving in place of “geographical” or “racial” names giving.
2. Will it be a good policy that henceforth, before accepting for publication, editors insist authors change any such names after cities and countries?
  Yes, they must, especially when they scent it can have fallout where peoples and races can be legitimately incensed, and names giving smells of names calling.
3. What should editors do so that corresponding authors ensure concurrence from all authors, especially the lead author?

Obtain written permission from all authors and especially the lead author (if he is not also the corresponding author) to the final draft of the paper before submission, or see to it that the corresponding author obtains it and submits it to them. This must be one of the preconditions to publication.

### Guidelines for editors in scientific names giving

The guidelines for editors in scientific names giving, therefore, are as follows:

- If any new name is given to a bacterium, enzyme, gene, disease, syndrome, etc. after a city, race, or country, simply reject the name and ask the researcher to give a scientific name based on the underlying reasons and mechanisms behind the disease, bacteria, gene, enzyme, syndromes, etc.
- If any name is given after a scientist or an institution, verify it is not self-naming, for that is bad form. Verify by peer review that the name is attributed to an accepted authority in the field, and preferably, get the authority’s concurrence, if living.
- If any other name is given, verify by peer review that it is scientific and factual as per the prevalent knowledge in the field.

## Concluding Remarks (see also Figure 1)

**Figure 1:**
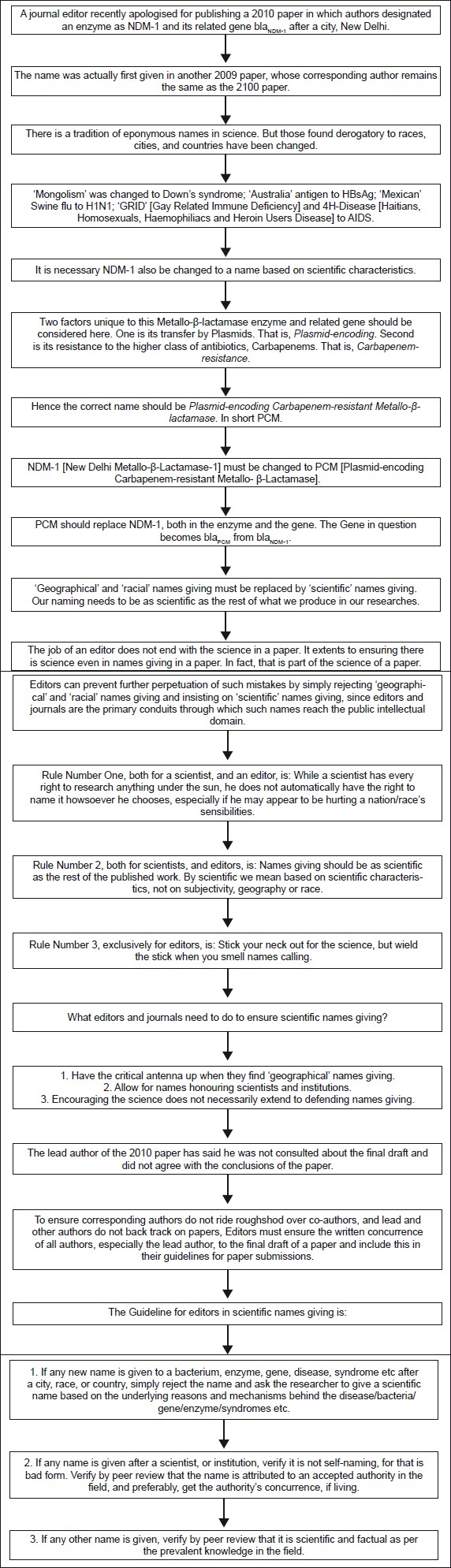
Flowchart of paper

The naming of an enzyme, gene, disease, etc. after a city, country or race is convenient but poor science. Naming should rather be based on scientific characteristics or in honour of the scientist/institution who/which pioneered it.

The name NDM-1 must be changed to PCM (New Delhi metallo-β-lactamase-1 must become plasmid-encoding carbapenem-resistant metallo-β-lactamase).

There is a tradition of naming diseases and organisms after cities, countries, scientists, and even races. There is an equally strong tradition of renaming such diseases and organisms when they are felt to be derogatory to such cities, countries and races.

Scientists who follow the first tradition are often unaware of the second. It is time they realise this and desist from this practice henceforth.

The tradition of eponymous naming is waning, but must be consigned to the dustbin of scientific history.

Journal editors must lay down in their guidelines for paper submissions the following:

1. Organisms, genes, bacteria, etc. cannot be named after cities, countries and races. They must be named according to their scientific characteristics.
2. All authors, and especially the lead author, read and agree to the final draft of a paper, especially when the corresponding author is not also the lead author.

The bottom line for editors and scientists is:

Follow scientific principles even in names giving. While a scientist has every right to research anything under the sun, he does not automatically have the right to name it howsoever he chooses, especially if he may appear to be hurting a nation/race’s sensibilities. An editor must ensure this is complied with in his journal.

The bottom line exclusively for an editor is:

Stick your neck out for the science, but wield the stick when you smell names calling.

All such names still in circulation need to undergo review and must be changed based either on their scientific characteristics or on the scientist/institution who/which first discovered them.

### Take Home Message

1. Change NDM-1 to PCM.
2. Stop naming organisms, genes, bacteria, diseases after cities, countries and races.
3. “Geographical” or “racial” names giving must be replaced by “scientific” names giving.
4. Journal editors must get written approval of all authors to the final draft of a paper, and not just of the corresponding author. Especially, the approval of the lead author should be got.
5. Journal editors must include items 3 and 4 in guidelines for paper submission.

## Questions That This Paper Raises

- Is it practical to carry out scientific names changing? Will it not result in a wild goose chase?
- Will not editors be burdened with greater responsibility? Also, will not shifting the onus on them make them come in the line of fire?
- Should we really consign eponymous naming to the dustbin of medical history?
- Corresponding authors and other authors better sort matters out amongst themselves. Why should editors fight their battles, or become quixotic knights rushing to the rescue of author ’damsels’ in distress?
- Should the author/s who first gave a certain name be the sole arbiters in a name change?
- There are many such names after cities and countries, but they are sportingly accepted by its citizens. Why can Indians not also be sporting and smile it off? After all, what’s in a name?
- Would it not be better India concentrate on improving its hygiene, sanitation and health facilities, and its indiscriminate antibiotic use, rather than raise the decibel by fighting phantasms of city names after microbes, genes and enzymes?

## About the Author


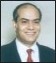
*Ajai R. Singh, M.D., is a Psychiatrist and Editor, Mens Sana Monographs (http://www.msmonographs.org). He has written extensively on issues related to psychiatry, philosophy, bioethical issues, medicine, and the pharmaceutical industry. © MSM*
